# The PIDDosome controls cardiomyocyte polyploidization during postnatal heart development

**DOI:** 10.1038/s41418-025-01645-x

**Published:** 2026-01-12

**Authors:** M. Leone, N. Kinz, F. Eichin, D. Obwegs, V. C. Sladky, V. Z. Braun, R. Hirschberger, D. Rizzotto, L. Englmaier, C. Manzl, K. Moos, J. Mergner, P. Giansanti, N. Martinez-Garcia, M. M. Marques, E. D. Jacotot, L. Eblahed, R. Yousif, M. K. Wright, D. Dawood, L. S. Maupome, C. Savko, M. Boerries, M. A. Sussman, A. Villunger

**Affiliations:** 1 Biocenter, Institute for Developmental Immunology, Medical University of Innsbruck, Innsbruck, Austria; 2 CeMM Research Center for Molecular Medicine of the Austrian Academy of Sciences, Vienna, Austria; 3 Institute of Neuropathology and Neuromolecularpathology, Medical University of Innsbruck, Innsbruck, Austria; 4 Institute of Medical Bioinformatics and Systems Medicine (IBSM), Medical Center – University of Freiburg, Faculty of Medicine, University of Freiburg, Freiburg, Germany; 5 TranslaTUM, Center for Translational Cancer Research, Technical University of Munich, Munich, Germany; 6 Bavarian Center for Biomolecular Mass Spectrometry at the University Hospital rechts der Isar BayBioMS@MRI, Technical University of Munich, Munich, Germany; 7 Instituto de Biomedicina and Departamento de Producción Animal, Universidad de León, León, Spain; 8 Inserm U1268, Medicinal Chemistry and Translational Research, Paris, France; 9 Faculté de Pharmacie, UMR 8038 CiTCoM, Université Paris Cité, Paris, France; 10 San Diego State University Heart Institute and Department of Biology, San Diego State University, San Diego, CA USA; 11 German Cancer Consortium (DKTK), Partner site Freiburg, A Partnership Between DKFZ and Medical Center - University of Freiburg, Freiburg, Germany

**Keywords:** Cell biology, Genetics

## Abstract

The adult mammalian heart is characterized by post-mitotic polyploid cardiomyocytes (CMs). Understanding how CMs regulate cell cycle exit and polyploidy can help developing new heart regenerative therapies. Here, we uncover that the PIDDosome, a multi-protein complex activating the endopeptidase Caspase-2, helps to implement a CM-specific differentiation program that limits ploidy during postnatal heart development. DNA content analyses show that cell-autonomous PIDDosome loss causes an increase in nuclear and cellular CM ploidy. Increased ploidy does not affect cardiac structure nor function in early adulthood, but correlates with a modest reduction in cardiac performance in aged mice. PIDDosome-imposed polyploidy control commences at postnatal day 7 (P7), reaching a plateau by P14. PIDDosome activation requires ANKRD26, targeting PIDD1 to mother centrioles. Opposite to prior observations in liver development, the PIDDosome limits CM polyploidization in a p53-independent manner but reliant on induction of *p21/Cdkn1a*, a notion supported by nuclear RNA sequencing and genetic deletion experiments. Our results provide new insights how proliferation of polyploid CMs is restricted during postnatal heart development.

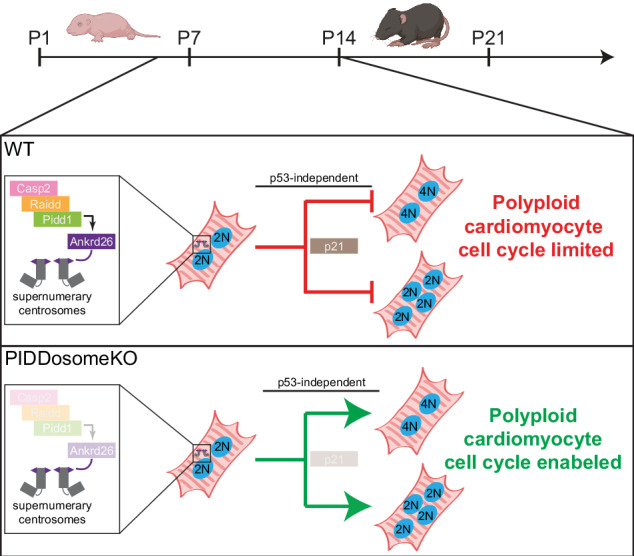

## Introduction

Ischemic heart disease (IHD) is one of the leading causes of mortality worldwide with nearly 9 million deaths documented in 2017 [1]. The primary cause of a reduced heart function is the initial loss of cardiomyocytes (CMs) and the inability of humans to compensate it [2, 3]. In order to reverse this loss, induction of proliferation in pre-existing CMs appears to be a promising approach but is still not applicable due to a clear gap of knowledge of the terminal differentiation process, activated postnatally in CMs. After birth, CMs undergo a decrease of cell cycle activity, which is coupled with a change of metabolism from glycolysis to fatty acid oxidation, and an increased maturation status of the sarcomere apparatus [4, 5]. Within the first postnatal week, in rodents, the majority of CMs fail cytokinesis during mitosis resulting in binucleated CMs [2, 6]. In the second to third postnatal week, a last wave of DNA synthesis occurs in a small subpopulation of binucleated CMs re-entering the cell cycle and thus, increasing nuclear ploidy [7]. Consequently, the adult mouse and rat heart consist of ~80–90% binucleated CMs, while the adult human heart contains an estimated ~25% binucleated and ~25–40% polyploid mononucleated CMs [3, 8–10]. Notably, recent studies showed that polyploidy limits cardiac regeneration by reducing the proliferation capacity of CMs [11, 12]. Therefore, a better understanding of CM polyploidization is of great interest to uncover new avenues to treat heart diseases.

A common feature of mono- and binucleated polyploid cells is the presence of supernumerary centrosomes [13]. The centrosome is formed by two centrioles, referred to as mother and daughter, respectively, surrounded by a pericentriolar matrix and acts as the major microtubule organizing center (MTOC) in most animal cells [14]. Mother centrioles differ from their daughters because they recruit distal appendage proteins, including ODF2, CEP83, SCLT1 and ANKRD26 [15]. Postnatal binucleated CMs show extra centrosomes. In fact, ~65% of postnatal day 3 (P3) binucleated CMs contain 4 centrioles, while ~27% have 3 centrioles, with 2 mother centrioles [16]. Moreover, once stimulated with pro-proliferative factors in vitro, P3 binucleated CM can re-enter the cell cycle resulting in the formation of either a binucleated cell or two mononucleated polyploid cells [16]. This shows that polyploid CMs have the intrinsic capability to cycle, but efforts to induce this process in adult CMs have largely failed due to the lack of knowledge of mechanisms controlling cell cycle exit after polyploidization.

Polyploid cells with extra centrosomes can undergo a p53-dependent cell cycle arrest or a cell death response [17, 18]. This is initiated by the “PIDDosome” [19], a multiprotein complex formed by the auto-processed form of PIDD1 (P53-induced death domain protein 1), termed PIDD1-CC, which recruits the bipartite adapter RAIDD/CRADD (RIP-Associated ICH1/CED3-homologous protein with Death Domain), and the pro-form of Caspase-2, a member of the family of cysteine-driven proteases known to control cell death and inflammation [20]. PIDD1 localization to the mother centrioles via the distal appendage protein ANKRD26, as well as centrosome clustering are requirements for the activation of this signaling complex, leading to Caspase-2 activation [21, 22]. Active Caspase-2 proteolytically inactivates MDM2, the master regulator of cellular p53 levels, and thereby stabilizes p53 protein to promote a p21-dependent cell cycle arrest [19].

Although first described in cancer cells forced to fail cytokinesis [19], the absence of either Caspase-2, RAIDD, or PIDD1, similar to p53- or p21-loss itself, increases ploidy in murine primary hepatocytes as part of a physiological polyploidization program during liver organogenesis [23]. Interestingly, the same study has shown that this function of the PIDDosome is preserved during liver regeneration, and likely also acts in human liver regeneration [23]. These findings raise the question of whether the PIDDosome and p53/p21 are also involved in controlling the scheduled polyploidization of CMs during postnatal heart development.

## Methods

### Animals

Generation and genotyping of *Casp2*^*−/−*^, *Casp2*^fl/fl^, *Raidd*^*−/−*^, *Pidd1*^*−/−*^*mT*/*mG*, *p53*^*−/−*^*, p73*^*−/−*^ and *p21*^*−/−*^ mice was previously described [24–30]. *XMLC2-Cre* (*XMLC2*^*+*^) mice were a kind gift from Prof. Felix Engel [31]. In order to generate *XMLC2*^*+*^*Casp2*^*fl/fl*^*mice*, *XMLC2*^*+*^ mice were crossed with the *Casp2*^*fl/fl*^ mice [25]. *XMLC2*^*+*^mice were crossed with a switchable Tomato and GFP reporter allele (mT/mG) in the Rosa26 locus, allowing expression of membrane bound versions of both fluorescent proteins. In order to generate *Casp2/p21*^*−/−*^ double knockout (dKO) mice, *Casp2*^*−/−*^ mice were crossed with *p21*^*−/−*^ mice. All strains used were maintained on a C57BL/6N background and housed at the Animal Facility of the Medical University of Innsbruck under specific pathogen free conditions. *Ankrd26*^*−/−*^ mice were generated, maintained and genotyped as previously described [32] and heart tissues from littermates were kindly provided by Prof. Andrew J. Holland. In all experiments, age-matched animals were used indiscriminate of sex.

### Echocardiography

Transthoracic echocardiography was performed on lightly anesthetized mice under isoflurane (1.0–2.0%; Abbot Laboratories) using a Vevo 2100 (VisualSonics). Hearts were imaged in the 2D parasternal short-axis (SAX) view, and M-mode echocardiography of the mid-ventricle was recorded at the level of papillary muscles. From the recorded M-mode images, the following parameters were measured: ejection fraction (EF), fractional shortening (FS), interventricular septum (IVS), LV posterior wall thickness (PWT), LV internal diameter (LVID), and LV volume in diastole (index: d) and systole (index: s).

### Adult and postnatal cardiomyocyte isolation

Adult hearts from 3-month-old animals of the indicated genotypes were sacrificed by CO2 asphyxiation and cervical dislocation according to the governmental and international guidelines on animal experimentation. Adult cardiomyocytes were isolated by following the protocol of a Langendorff-free method published by Ackers-Johnson and colleagues [33] with minor changes. 30 µM CaCl_2_ was freshly added to  the Collagenase buffer, to optimize the digestion step. In order to have a final concentration of 0.5 mg/ml, Collagenase II (255 units/mg, 17101-015, Life Technologies, USA) and Collagenase IV (310 units/mg, 17104-019, Life Technologies) were used at 16.52 mg and 16.81 mg, respectively, as the unit concentrations of these enzymes were different from the original protocol. Moreover, the freshly made collagenase buffer, was pre-warmed on a heating magnetic stirrer at 37 °C just before being injected in the left ventricle (LV). As first step, 7 ml of EDTA buffer was injected into the apex of the right ventricle with 1 ml/min speed as described in the original protocol. Then, 20 ml EDTA buffer was injected into the LV over 10 min, and subsequently 3 ml of Perfusion buffer was injected into the LV with 1 ml/min speed. 40 ml of Collagenase buffer was finally injected into the LV over 13 min. However, after 8 min the heart was examined to see whether it has lost its color and structure and if it appeared over-digested. If this was the case, the digestion step was stopped at 8 min. After heart digestion and creating ~1 mm^3^ pieces, the tissue trituration was done by gently pipetting for 2 min, followed by the addition of Stop buffer and further gentle pipetting for another 4 min. Adult cardiomyocyte vitality was checked after each isolation via Trypan blue staining. In order to assess cellular ploidy, cells were then centrifuged for 2 min at 100 × *g*, fixed in 4%PFA diluted in PBS for 20 min at RT and then washed 3 times with PBS.

Hearts from postnatal day 1 (P1), P7 and P10 mice from the C57BL/6N mouse strain were dissected upon decapitation with operating scissors (RS-6845, ROBOZ, USA), the atria were removed, and the remaining ventricles were minced. For the isolation of postnatal cardiomyocytes, mouse hearts were isolated and digested utilizing the gentleMACS Dissociation kit (130-098-373, Milteny Biotech GmbH, Germany) according to the manufacturer’s instructions. For cardiomyocyte enrichment, cells were pre-plated in DMEM-F12/Glutamax TM-I (10565, Life Technologies)/10% fetal bovine serum (FBS, F0804, Sigma-Aldrich, USA)/penicillin (100 U/ml)/streptomycin (100 µg/ml) (Pen/Strep, P0781, Sigma-Aldrich). After 1.5 h, non-attached cells, enriched in cardiomyocytes, were collected, centrifuged for 10 min at 300 × *g*, resuspended in DMEM-F12, Glutamax TM-I containing 3 mM Na-pyruvate, 0.2% BSA, 0.1 mM ascorbic acid, 0.5% Insulin-Transferrin-Selenium (100x, 41400045, Life Technologies), 1% FBS, penicillin/streptomycin (100 U/mg/ml) and counted. Postnatal cardiomyocytes were washed once with PBS (14190169, Thermo Fisher Scientific, USA) and the cell pellet was snap-frozen in liquid nitrogen to preserve RNAs.

### Cardiomyocyte nuclei isolation and flow cytometric analyses

The isolation of CM nuclei was performed by following the previously described protocol [34, 35] with minor changes. Snap-frozen ventricles from selected genotypes were cut in 1 mm^3^ pieces and transferred into a falcon containing 15 ml of lysis buffer and then, homogenized with a TP 18/10 Ultra-Turrax probe homogenizer (IKA, Germany) at 20,000 rpm for 20 s. In order to isolate the CM nuclei, the 15 ml lysis buffer solution with the homogenized tissues was passed 8 times up-and -down through a 20 ml syringe with a 20 G needle. The crude nuclei isolate was filtered using at first, a 100 µm cell strainer, and subsequently, a 70 µm cell strainer. After centrifugation for 10 min at 700 × *g* at 4 °C, the nuclei pellet was dissolved in 5 ml sucrose buffer and then, topped up to 20 ml total solution. 10 ml sucrose buffer was added into a 1% BSA/PBS pre-coated ultra-centrifuge tube and was overlayed by the 20 ml nuclei suspension before spinning for 60 min at 13,000 × *g* at 4 °C. After centrifugation, the sucrose buffer and any remaining liquid were quickly removed and the nuclei at the bottom of the tube were resuspended in 700 µl of nuclei storage buffer. Next, 600 µl of nuclei solution was incubated with a rabbit anti-PCM1 antibody (1:300, HPA023370, lot. number: 000007967, Sigma-Aldrich) overnight at 4 °C with constant shaking. 100 µl of the nuclei solution was used for the negative control of the staining. On the next day, both nuclei samples were washed by adding 2 ml of PBS, centrifuged for 10 min at 700 × *g* at 4 °C and then, the nuclei pellets were incubated with goat anti-rabbit Alexa 647-conjugated antibodies (1:500, Life Technologies) for 60 min at 4 °C. After 1 h, the samples were washed with PBS and incubated with Propidium Iodide (1:25 of a 1 mg/ml solution). The gating strategy for ploidy analysis is shown in Supplementary Fig. 1A, nuclear DNA content was measured on a flow cytometer (LSR-Fortessa, BD Biosystems, USA) and data were analyzed quantitatively, excluding doublets, using FlowJo (FlowJo X, LLC).

### Histology and immunohistochemistry

In order to have cardiomyocytes in a relax status (diastole), hearts were injected with a cardioplegic solution (25 mmol/L KCl) in the LV. Once the hearts stopped beating, they were cut from the aorta branch and placed in 4% PFA diluted in PBS overnight at RT. On the next day the fixed hearts were dehydrated and paraffinized. For hematoxylin and eosin staining, 4 µm coronal heart sections were deparaffinized, and rehydrated using graded xylol and ethanol incubation steps and then stained. At least 6 different hearts per genotype were analyzed. For detection of cell death, 4 µm coronal heart sections were deparaffinized and rehydrated. TUNEL in situ nick end-labeling of DNA was performed following manufacture’s instruction (#G7132, DeadEnd Colorimetric TUNEL System, Promega). The nuclei were counterstained with hemalum solution (#T865.3, Carl ROTH, Germany). After staining, sections were mounted and images acquired on a NanoZoomer S210 Digital slide scanner (C13239-01, Hamamatsu photonics, Japan).

For collagen staining, formalin-fixed, paraffin-embedded (FPPE) hearts, sectioned in the coronal orientation were stained with picrosirius red and fast green with the following protocol. Slides were deparaffinized and rehydrated with water then stained with 0.4% fast green for 15 min. Slides were rinsed with water and stained with 0.1% picrosirius red for 1 h. Slides were rinsed with 0.5% acidified water, dehydrated with 100% alcohol then xylene, and mounted in ClearVue (Thermo Fischer Scientific). Red= collagen. Green = non-collagen proteins. Stained hearts were imaged on the Keyence microscope at 20X magnification. 8 set points around the perimeter of the heart were used as boundaries and to set the autofocus map. Each individual sample was white balanced and captured with 1/250 s exposure. Tile scans were acquired using the Keyence stitching software and exported uncompressed. Tile scans were analyzed in ImageJ for collagen quantification and total area measurements. The scale bar was used to set the micron scale for each individual tile scan. Atria, valves, and artifacts were removed using the “clear” tool in ImageJ. The whole heart area was measured using the color thresholding tool with the hue at 0–255, the saturation at 15–255, and brightness at 10–255. The red hue resulting from the picrosirius red stain was measured at the hue of 200–255 with the saturation and brightness the same as stated above. The percentage of collagen deposition was calculated as collagen area divided by total heart area multiplied by 100.

### Cryosections

Hearts from mentioned genotypes were pre-fixed in 4% PFA overnight at 4 °C and on the next day, washed 3 times with PBS and incubated in 15% sucrose diluted in MilliQ water for 4 h at 4 °C. After the 15% Sucrose solution, hearts were submerged in 30% sucrose diluted in MilliQ water overnight at 4 °C. Afterwards, hearts were embedded in Tissue-Tek O.C.T. compound tissue-freezing medium (4583, Sekura, Germany), frozen in liquid nitrogen, and sectioned with a Microm HM550 (Thermo Fischer Scientific) (10 µm). Once the tissues were cut, the sections were left to better adhere on the slide for 30 min at RT.

### Immunofluorescence analyses

Immunostainings were performed as previously described [36], but after washing and incubation in fresh staining solution without antibodies, the CM suspensions were centrifuged for 2 min at 100 × *g* at RT. Before antibody incubation, freshly isolated and PFA-fixed CMs were permeabilized with 0.5% Triton X-100/PBS for 10 min at RT. Primary antibodies: rabbit anti-Pan cadherin (1:100, C3678, Sigma-Aldrich,), mouse anti-sarcomeric-µ-actinin (1:200, ab9465, Abcam). After overnight incubation, primary antibodies were detected by using Alexa Fluor™ 488, Alexa Fluor™ 594 or Alexa Fluor™ 647 conjugated antibodies (1:500, Life Technologies, USA). DNA was visualized with SYTOX™ Green Nucleic Acid Stain (1:200,000 in PBS with 0.1% triton and 10% horse serum, S7020, Thermo Fisher Scientific). Samples were diluted in PBS in order to acquire pictures. Confocal images were captured on a spinning disk confocal laser scanning microscope (Cell Voyager CV1000, Yokogawa, Tokyo, Japan).

FFPE heart sections were boiled in antigen retrieval buffer (1 mM EDTA, pH 8.0) for 8 min in a microwave (700 W). After the antigen retrieval step, slides were kept for 30 min at RT and rinsed 3 times in PBS. Next, the slides were incubated in WGA (1:100 diluted in PBS, W11261, Thermo Fisher Scientific,) for 10 min at RT and then, washed 2 times with PBS. After this step, immunostainings were performed as previously described [36]. As primary antibody a goat anti-Troponin I (1:500, ab56357, Abcam) was used, which was detected by an Alexa Fluor™ 594 conjugated antibody (1:500, Life Technologies, USA). DNA was visualized with 0.5 µg/ml DAPI (4′,6′-diamidino-2-phenylindole) (Sigma Aldrich). Samples were mounted with Fluoromount-G™ (Thermo Fisher Scientific). Images were captured on a Zeiss Axiovert 200 M microscope using the VisiView 4.1.0.3 (Visitron Systems) acquisition software.

Immunostainings of pre-fixed heart cryosections were permeabilized with 0.5% Triton in PBS for 10 min at RT. Subsequently, the above-mentioned immunostaining protocol [36] was used with a minor modification of the time of secondary antibody incubation, set to 1 h at RT. High resolution images were captured on a LSM980 confocal laser scanning microscope (ZEISS, Germany).

### RNA isolation and qRT-PCR

Postnatal CM pellets were processed in order to extract total RNA using TRIzol TM Reagent (15596018, Invitrogen, USA) according to the manufacturer. 1 µg of total RNA was used for generation of cDNA (iScript cDNA synthesis kit, 170–8891, BioRad, USA). For quantitative real-time PCR (RT-qPCR) experiments 140 ng of cDNA per each reaction was used. RT-qPCR assays were performed in experimental triplicates for each biological replicate using Luna® Universal Probe One-Step RT-qPCR Kit (E3006, New England biolabs, USA) in a StepOne Plus real time PCR system (Applied Biosystems, USA). Relative gene expression was calculated based on ΔCt values using *mGapdh* as housekeeping gene.

### Bulk nuclear RNA sequencing (RNAseq) pre-processing and analysis

For extraction of nuclear CM RNA, postnatal cardiomyocyte nuclei were isolated and stained using anti-PCM1, as described above. Subsequently, cardiomyocyte nuclei were sorted based on their PCM1 staining by using a BD FACSAria™ III Cell Sorter (648282, BD Bioscience). Importantly, in order to obtain enough RNA material from the isolated cardiomyocyte nuclei, several hearts from the same postnatal days were pooled together per biological replicate (P1: 15 hearts, P7: 6 hearts, P14: 2 hearts). Afterwards, the low-binding Eppendorf tubes containing the sorted cardiomyocyte nuclei were centrifuged for 10 min at 700 × *g* at 4 °C. The resulting nuclei pellets were processed for RNA extraction by utilizing the Quick-RNA MicroPrep Kit (R1050, Zymo Research, USA) following the manufacturer’s protocol. RNA-sequencing library were prepared by Lexogen NGS Services (Vienna, Austria) using the QuantSeq 3′ mRNA-Seq Library Prep Kit FWD for Illumina and following the low-input protocol. Sequencing was performed on an Illumina NextSeq 2000 at Lexogen NGS Services to produce 100 bp single-end reads for each sample. Raw RNA sequencing reads were quality-controlled with FastQC (v0.11.8) [37] and preprocessed with cutadapt (v4.0) [38] to trim poly-G stretches resembling sequencing artefacts, trim low-quality bases from the 3’end, trim adapter and poly-A sequences introduced by the sequencing strategy, remove low-quality reads (more than 1 expected error and/or more than 30% N-bases) and remove short reads (less than 20 nucleotides). Processed reads were aligned against the mouse reference genome GRCm39 from Ensembl (v108) [39] using STAR (v2.6.1e) [40]. All following analyses were performed within R v. 4.2.1. The number of reads per gene (considering the full gene, i.e. both exonic and intronic sequences) was counted with HTSeq (v2.0.3) [41]. Gene count normalization and differential gene expression analysis were performed with the R package limma (v. 3.52.4) [42]. Genes were considered as significantly differentially expressed with an adjusted *p*-value < 0.05 and an absolute log2 fold change >1. Gene set enrichment analysis was carried out using the R package gage (v. 2.46.1) [43], based on a selection of gene sets retrieved with the R package msigdbr (v. 7.5.1) [44] (included gene set collections: hallmark, canonical pathways excluding WikiPathways, transcription factor targets, Gene Ontology). Gene sets were considered as differentially expressed when presenting with a plain *p*-value < 0.05.

Before gene count normalization, differential gene expression analysis, and gene set enrichment analysis, three samples (P1 *XMLC2*^*-*^*Casp2*^*fl/fl*^ replicate 1, P14 *XMLC2*^*-*^*Casp2*^*fl/fl*^ replicate 3, P14 *XMLC*^*+*^*Casp2*^*fl/fl*^ replicate 1) were excluded due to low quality. The number of detected genes (at least one read count) was particularly low for P14 *XMLC2*^*-*^*Casp2*^*fl/fl*^ replicate 3 (1676 genes) and P14 *XMLC*^*+*^*Casp2*^*fl/fl*^ replicate 1 (3893 genes) compared to the other samples (between 7223 and 17057 genes) (data not shown). P1 *XMLC2*^*-*^*Casp2*^*fl/fl*^ replicate 1 clustered particularly far away in a Principal component analysis (PCA) of all samples (PCA calculated with R package labdsv v2.1-0 [45] (Suppl. Figure 3A). A fuzzy clustering of all samples showed clusters of genes (clusters 6 and 7) with high expression level in P1 *XMLC2*^*-*^*Casp2*^*fl/fl*^ replicate 1 compared to replicates 2, 3, and 4 (fuzzy clustering performed with R package Mfuzz v2.56.0 [46] (Supplementary Fig. 4). The clustering was calculated on the gene log2 fold changes with respect to the average gene counts of the four P1 *XMLC2*^*-*^*Casp2*^*fl/fl*^ replicates, after each sample’s raw counts were normalized with the average count of its interquartile range count values. Subsequent functional enrichment analysis of gene cluster 6 and cluster 7 returned significantly enriched gene sets (adjusted *p*-value < 0.05) associated with mitochondria, ribosomes and translation (Supplementary Fig. 3B). Functional enrichment was calculated with Fisher’s exact test, based on the same gene set collection as described above. As these results are indicative of a cytoplasmic contamination of P1 *XMLC2*^*-*^*Casp2*^*fl/fl*^ replicate 1, this sample was not considered for further analysis. GEO submission number (GSE275946). Reviewer token (uvyhckkqxvwfvap).

### Tissue lysis and SP3 proteome clean up

Pulverized tissues from P7 hearts of *XMLC2*^*-*^*Casp2*^*fl/fl*^ and *XMLC*^*+*^*Casp2*^*fl/fl*^ mice were lysed, reduced, and alkylated by heating for 10 min at 95 °C under shaking at 1500 rpm in lysis buffer containing 6 M guanidine hydrochloride (GuHCl, Sigma), 10 mM tris(2-carboxyethyl) phosphinehydrochloride (TCEP, Thermo Fisher), 40 mM chloroacetamide (CAA, Sigma), and 200 mM 3-[4-(2-Hydroxyethyl)piperazin-1-yl]propane-1-sulfonic acid (HEPPS, Sigma), pH 8.5. Protein concentration was estimated using a BCA assay (Thermo Fisher). For each sample, 200 μg of protein were incubated with 10 μL of SP3 beads [47] (1:1 mix of Sera-Mag Speed Beads A and B, Cytiva). Pure ethanol (EtOH, VWR) was added to achieve a final concentration of 80% (v/v), and samples were incubated at room temperature in a thermoshaker for 18 min at 800 rpm. The samples were then placed on a magnetic rack for 2 min to immobilize the SP3 beads, after which the supernatant was discarded. The beads were washed twice with 1 mL of 80% (v/v) EtOH in water and once with 800 µL of acetonitrile (ACN, VWR).

### TMT labeling and protein digestion

SP3-bound proteins were resolubilized with 120 μL of labeling buffer containing 2 M GuHCl, 10 mM TCEP, and 200 mM HEPPS, pH 8.5. The samples were incubated at room temperature in a thermoshaker for 30 min at 800 rpm, with 1 min sonication in a water bath every 10 min. TMT10-plex labeling (Thermo Fisher) was performed as previously described [48]. Briefly, 0.8 mg of TMT reagent dissolved in 30 µL of 100% anhydrous ACN (Sigma) was added to the samples to label free N-termini (protein N-termini and lysine residues). The labeling reaction proceeded for 1 h at 20 °C in a thermoshaker at 400 rpm. The reaction was quenched by adding 5 µL of 1 M Tris/HCl pH 8.5 (Sigma) and incubating for 30 min at 25 °C. After quenching, the samples were combined, and SP3 cleanup was repeated to remove excess reactants as described above. The combined proteome was resuspended in 100 mM 4-(2-hydroxyethyl)-1-piperazineethanesulfonic acid (HEPES, Sigma), pH 7.6, and digested overnight at 37 °C with trypsin at 1:50 protease:protein ratio (w/w).

### N-terminome negative selection

To remove internal trypsin-generated peptides, the digest was incubated overnight with 30 mM sodium cyanoborohydride (Sigma) and the amine-reactive hyperbranched aldehyde-derivatized polymer [49] (HPG-ALDII https://ubc.flintbox.com/technologies/888fc51c-36c0-40dc-a5c9-0f176ba68293) at 1:5 peptide:polymer ratio (w/w). The reaction was quenched by adding Tris/HCl pH 7.6 to a final concentration of 100 mM. The resulting sample was centrifuged using an Amicon 30 kDa filter (Sigma) to collect TMT-labeled N-termini in the flow-through. The TAILS sample was acidified to 1% formic acid (FA, Carlo Erba), desalted and further fractionated into six fractions using the high pH RP fractionation [50], employing a self-packed StageTip with five disks of C18 material (3 M Empore). The peptides were eluted with 25 mM ammonium formate pH 10 (Sigma), using increasing concentrations of ACN (5, 7.5, 10, 12.5, 15, 17.5, and 50%). Finally, the seven fractions with flow-through were combined into six fractions (5  +  50%, 7.5%, 10%, 12.5%, 15%, and 17.50% + flow-through), the peptides were dried in vacuo and stored at −20 °C until further use.

### nLC-MS/MS

Nano flow LC-MS/MS measurements were performed using a Dionex Ultimate 3000 UHPLC+ system coupled with an Orbitrap Eclipse mass spectrometer (Thermo Fisher). Peptides were delivered to a trap column (75 μm i.d. × 2 cm, packed in-house with 5 μm Reprosil C18 beads, Dr. Maisch) and washed using 0.1% FA at a flow rate of 5 μL/min for 10 min. Subsequently, peptides were transferred to an analytical column (75 μm i.d. × 40 cm, packed in-house with 1.9 μm Reprosil C18 beads, Dr. Maisch) at a flow rate of 300 nL/min. Peptides were chromatographically separated using an 80 min linear gradient from 8 to 34% of solvent B (0.1% FA, 5% DMSO (Sigma) in ACN and solvent A (0.1% FA, 5% DMSO in water).

The Orbitrap Eclipse was operated in a data-dependent acquisition (DDA) to automatically switch between MS and MS/MS. Briefly, survey full-scan MS spectra were recorded in the Orbitrap from m/z 360 to 1500 at a resolution of 60 K, using an automatic gain control (AGC) target value of 100% and maximum injection time (maxIT) of 50 ms. For the MS3-based TMT method, initial MS2 spectra for peptide identification were recorded in the Orbitrap at a resolution of 15 K with a top speed approach using a 3-s duration (isolation window m/z 0.7, AGC target value of 100%, maxIT of 22 ms). Fragmentation was set to HCD, with a NCE of 34%. Then, for each peptide precursor, an additional MS3 spectrum for TMT quantification was obtained in the Orbitrap at 30 K resolution with enhanced resolution mode enabled (AGC of 500%, maxIT of 54 ms). The precursor was fragmented as for the MS2 analysis, followed by synchronous selection of the 10 most intense peptide fragments and further fragmentation via HCD using a NCE of 55%. Dynamic exclusion was set to 90 s.

### Data analysis of N-terminomics

Raw mass spectrometry data were processed using the FragPipe software (version 21.1) with its built-in search engine MSFragger version 4.0 [51]. Spectra were searched against the mouse UniProtKB database UP00000589 (63,300 entries including isoforms, downloaded on 04.2024). Default parameters for a TMT10-MS3 search were employed, with a defined precursor tolerance of 20 ppm and enzyme semi-tryptic specificity for database digest set to trypsin_r. Methionine oxidation (+15.9949) and protein n-termini acetylation (+42.0106) were set to variable modifications. Cysteine carbamidomethylation (+57.02146), and TMT (+229.16293) on lysine and peptide n-termini were added as static modifications.

After peptide-to-spectrum matches (PSM) rescoring via percolator [52], identifications were adjusted to 1% false discovery rate (FDR) at the protein, peptide and PSM levels. TMT integrator, included in FragPipe, was used to perform MS3-based quantification of the detected peptide features, using default settings.

Data analysis was performed with the Perseus software (version 2.0.10.0.) [53]. Peptide identifications were filtered to remove contaminants before performing data normalization of the log2-transformed TMT intensity values by median centering, as implemented in Perseus. For statistical analysis, only peptides that had been quantified in at least 3 biological replicates were retained, and missing values were imputed from the normal distribution in Perseus, using default parameters. Potential caspase-2 substrates enriched in the WT over the KO samples were identified using the Significance A test [54] on the distribution of TMT ratios, using a Benjamini-Hochberg FDR threshold of 5%.

The mass spectrometry proteomics data have been deposited in the ProteomeXchange Consortium via the PRIDE partner repository [55] with the dataset identifier PXD060421 (Website: http://www.ebi.ac.uk/pride, Username: reviewer_pxd060421@ebi.ac.uk, Password: BHrAjVHxayye).

### Imaris image analysis

Nuclear and cellular ploidy measurements were performed by following the indications published by Bensley and colleagues [10]. More in detail, firstly, reconstituting the individual nuclei by the 3D volume module (“3D View” in Imaris) was accomplished and then, analyzing the mean intensity of the Sytox Green was achieved by using the “surface” Imaris software package. Importantly, since signal intensity is fundamental for accurate ploidy measurement, the same setting of laser power, voltage, offset, and pinhole across the board was kept constant for all the experiments. Single nuclei from binucleated CMs were taken as reference for diploid nuclei in this analysis. For nuclear and cellular ploidy analysis, images were captured on a spinning disk confocal microscope, CV1000 Cell voyager (Yokogawa, Japan).

### Statistical analysis

Data are expressed as the mean ± standard deviation (SD) or standard error of the mean (SEM) of at least three independent experiments if not stated otherwise. Statistical significance of differences was evaluated by either Student’s *t* test, or 1way or 2way ANOVA followed by Bonferroni’s post-hoc test, Sidak’s or Tukey’s multiple comparisons test (GraphPad Prism 9.0). *p* < 0.05 was considered as statistically significant. Further information on statistical testing and number of replicates used in individual experiments can be found in the figure legends.

## Results

### The PIDDosome controls CM ploidy

**Fig. 1:**
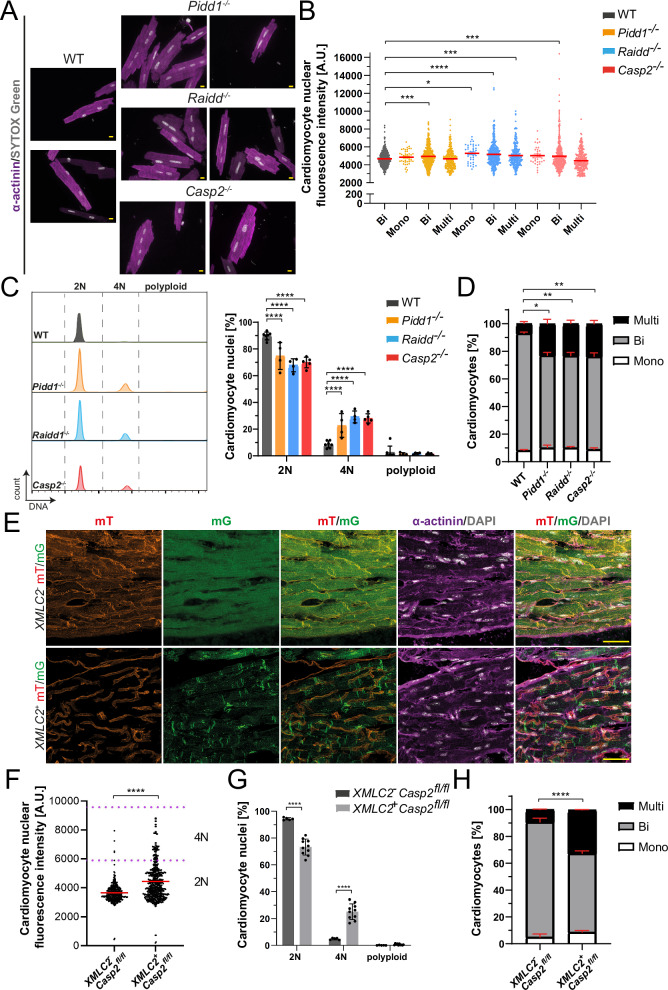
The PIDDosome regulates nuclear and cellular polyploidization of CMs. **A**, **B** Ploidy measurements of single CM nuclei by 3D volumetric analysis of Sytox Green fluorescence intensity in correlation with the cellular ploidy status. **A** Representative pictures of CMs freshly-isolated from 3-month-old mice of the indicated  genotypes. CMs were stained for the cardiac marker, α-sarcomeric actinin (purple), and a nuclear marker, SytoxGreen (gray). Scale bars: 10 μm. **B** Scatter blots of quantification of Figure A. n: 4 hearts per genotype; 100 CMs per heart. **C** Representative histograms (left) and quantification (right) of the CM nuclear ploidy measured by flow cytometry hearts from 3-month-old mice of the indicated genotypes. N: 7 (WT), n:4 (*Pidd1*^*−/−*^), n:4 (*Raidd*^*−/−*^), n:5 (*Casp2*^*−/−*^). **D** Bar graphs of CM cellular ploidy of the indicated genotypes at 3months of age. N: 4 hearts per genotype. 150 CMs per heart were quantified. Statistical analysis between the multinucleated group from the different genotypes is shown in the graph. **E** Validation of the cardio-specificity of the *XMLC2* promoter. Confocal pictures of cryosections of pre-fixed hearts stained for α-sarcomeric-actinin (purple) and DAPI (gray). Scale bars: 40 μm. **F** Quantification of CM nuclear ploidy by 3D volumetric analysis of Sytox Green fluorescence intensity. n: 3 hearts per genotype. 100 CM nuclei per heart were analyzed. **G** Quantification of CM nuclear ploidy of the indicated genotypes of 3-month-old mice by flow cytometry. n: 4 (*XMLC2*^-^ *Casp2*^*fl/fl*^), n: 8 (*XMLC2*^+^ *Casp2*^*fl/fl*^). **H** Bar plots of CM cellular ploidy of the indicated genotypes at 3months of age. n: 3 hearts per genotype; 150 CM nuclei per hearts were analyzed. Data represent means ± SEM (**B**, **D**, **F**, **H**) or ± SD (**C**, **G**) analyzed by One-way ANOVA (**B**) or Two-way ANOVA (**C**, **D**, **G**, **H**) or Two-tailed Student’s *t* test (**F**). *p < 0.05, **p < 0.01, ***p < 0.001, ****p < 0.0001, n.s. not significant.

First, we have quantified the nuclear ploidy levels of freshly-isolated CMs from 3-month-old mice lacking individual PIDDosome components (Fig. 1A). Ploidy of single CM nuclei was assessed by 3D volumetric analysis of Sytox Green fluorescence intensity and correlated with the cellular ploidy status. Moreover, the average nuclear staining intensity of single nuclei from binucleated CMs was used to define 2N ploidy, since more than 90% of binucleated CMs have diploid nuclei [10]. This analysis revealed that CM nuclei of binucleated cells from all three different PIDDosome knockout strains contain a higher nuclear ploidy, compared to nuclei from binucleated CMs from WT hearts. This indicates that binucleated CMs in *Pidd1, Raidd* and *Casp2-*deficient animals harbor an increased number of nuclei with DNA content >2N (Fig. 1B). To determine the exact nuclear ploidy of single CM nuclei from PIDDosome-mutant animals, we used a flow cytometry-based strategy. To discriminate CM nuclei from non-myocyte nuclei, we employed PCM1 staining [56] (Supplementary Fig. 1A). Intriguingly, PIDDosome-mutant animals showed a more than two-fold increase in the percentage of tetraploid (4N) CM nuclei, compared to those isolated from WT animals (Fig. 1C). Quantification of the cellular ploidy status of CMs showed that absence of the PIDDosome also causes a significant increase in multinucleated CMs (Fig. 1D). Altogether, these results indicate that abrogation of the PIDDosome induces a pronounced increase in nuclear, as well as cellular polyploidization of CM, likely due to tetraploid binucleated CMs entering another round of cell cycle, characterized by cytokinesis failure. This generates either octoploid binucleated CMs or multinucleated CMs with individual diploid nuclei.

To test if the observed phenotype in PIDDosome knockout mice is cell autonomous, we exploited cardiac specific deletion of *Casp2* (named from now on *XMLC2*^*+*^*Casp2*^*fl/fl*^) (Supplementary Fig. 1B). To confirm cardiac-specific CRE expression, *XMLC2-Cre* (*XMLC2*^*+*^), mice were first crossed with a fluorescence reporter line, carrying a switchable Tomato/GFP reporter allele in the *Rosa26* locus (*mT/mG*). In the *mT/mG* model, all cells initially express the Tomato reporter, but not GFP, which relies on CRE recombination (Supplementary Fig. 1C). Consistently, upon *XMLC2*-driven CRE activation, CM membranes in heart cryosections were GFP-positive, while non-CM membranes remained Tomato-positive. Staining with α-sarcomeric actinin was performed to define cell identity, confirming specificity and effective CRE-recombination (Fig. 1E). Importantly, measuring the CM nuclear ploidy in *XMLC2*^*+*^ mice, excluded off-target effects on nuclear ploidy (Supplementary Fig. 1D). 3D volumetric analysis of Sytox Green staining intensity, as well as flow cytometric analysis revealed that cardiac-specific *Casp2* deletion in *XMLC2*^*+*^*Casp2*^*fl/fl*^ mice significantly increased the percentage of polyploid 4N CM nuclei, when compared to *Casp2*^*fl/fl*^ control mice (*XMLC2*^*-*^*Casp2*^fl/fl^) (Fig. 1F, G). In addition, the cellular ploidy of *XMLC2*^+^*Casp2*^*fl/fl*^ CMs (Fig. 1H) increased to a similar degree as seen in *Casp2*^−/−^ animals (Fig. 1D).

Taken together, these data indicate that the PIDDosome controls CM polyploidization, limiting not only the nuclear but also cellular ploidy in a cell autonomous manner.

### Increased CM ploidy does not affect cardiac structure nor function in early adulthood

**Fig. 2:**
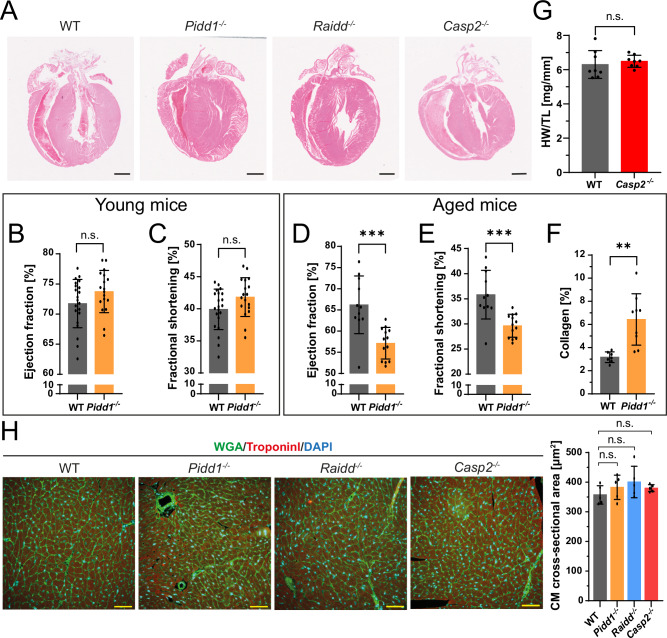
Heart function is not influenced by elevated CM ploidy in young but declines in aged mice. **A** Representative images of hematoxylin and eosin (H&E) stained FFPE heart sections of 3-month-old mice of the indicated genotypes. Scale bars: 1 mm. Quantification of the cardiac functions, ejection fraction (**B**) and fractional shortening (**C**), measured by echocardiography in 7–10-weeks-old mice. n: 21 (WT), n: 18 (*Pidd1*^−/−^). **D**, **E** Quantification of the cardiac functions, ejection fraction and fractional shortening in 15–19-months-old mice. n: 11 (WT), n: 13 (*Pidd1*^−/−^). **F** Quantification of collagen area in 15–19-months-old mice from WT (n:7) and *Pidd1*^*−/−*^ (n:9) mice. **G** Heart weight to tibia length (HW/TL) ratio of 3-month-old mice. n:8 (WT and *Casp2*^*−/−*^*)*. **H** Representative pictures (left) and cross-sectional area quantifications (right) of FFPE hearts from WT, *Pidd1*^−/−^, *Raidd*^−/−^ or *Casp2*^−/−^ mice stained for the membrane marker WGA (green), the cardiac marker Troponin I (red) and DAPI (blue) for the nuclei. Scale Bars: 50 µm. n: 5 (WT, *Pidd1*^−/−^, *Raidd*^−/−^, *Casp2*^−/−^, ~2100 CMs distributed within 7 pictures per heart were quantified). Data represent means ± SD (**B**–**H**) analyzed by Student’s *t* test (**B**–**G**) or One-way ANOVA (**H**). **p < 0.01, ***p < 0.001, n.s. not significant.

To test cardiac function, the heart ultra-structure of the different genotypes was analyzed by hematoxylin and eosin (H&E) staining on formalin-fixed and paraffin-embedded (FFPE) heart sections. No obvious differences were observed in *Casp2*^*−/−*^, *Raidd*^*−/−*^ or *Pidd1*^*−/−*^ hearts compared to WT hearts (Fig. 2A), nor in *XMLC2*^+^ *Casp2*^*fl/fl*^ *vs*. *XMLC2*^-^ *Casp2*^*fl/fl*^ hearts (Supplementary Fig. 1E). Likewise, echocardiography of 7–10 week-old mice lacking *Pidd1* suggested that the observed increase in the polyploid CM population did not affect the cardiac function. In fact, there were no significant differences in ejection fraction (EF) and fractional shortening (FS) between *Pidd1*^*−/−*^ and WT mice (Fig. 2B, C, Supplementary Fig. 1F). Of note, aged mice lacking *Pidd1* showed a mild but significant impairment of heart function, characterized by reduced EF as well as FS (Fig. 2D, E, Supplementary Fig. 1G). Moreover, signs of tissue remodeling, defined by an increase of collagen depositions, were noted in aged *Pidd1*^*−/−*^ hearts (Fig. 2F, Supplementary Fig. 1H).

As polyploid cells usually show increases in nuclear and cellular size [57], we investigated whether the observed ploidy increase affects heart weight. Loss of *Casp2* did not affect organ weight, as shown by the ratio between heart weight (HW) and tibia length (TL) (Fig. 2G). Consistently, cardiac hypertrophy in young adult mice was excluded as the diastole left ventricle volume, LV Vol;d, in *Pidd1*^−/−^ knockout was comparable to that of age-matched controls (Supplementary Fig. 1F). Yet, an increase in CM ploidy may not affect heart weight, but could still affect cell size. Quantification of the cross-sectional area of CMs from *Casp2*^*−/−*^, *Raidd*^*−/−*^*, Pidd1*^*−/−*^ and WT hearts, measured by Wheat Germ Agglutinin (WGA), staining cell membranes, and Troponin I, staining CMs, revealed no differences across genotypes (Fig. 2H). This suggests that the increases in ploidy are not associated with an increase in CM size, even though contrasting findings were made in the liver [58], at least in early life. To test if the observed absence of CM hypertrophy could be explained by increased polyploid CM cell death, TUNEL staining was performed on sections of 3 months-old PIDDosome knockout hearts. The assay showed no sign of increased apoptosis compared to WT (Supplementary Fig. 2A), excluding this possibility.

Collectively, these data show that increases in the number of polyploid CM do not alter the tissue architecture of the heart and more importantly, cardiac function in young adult mice. However, these increases appear to reduce cardiac function with age.

### The PIDDosome restricts CM ploidy in response to  extra centrosomes

**Fig. 3:**
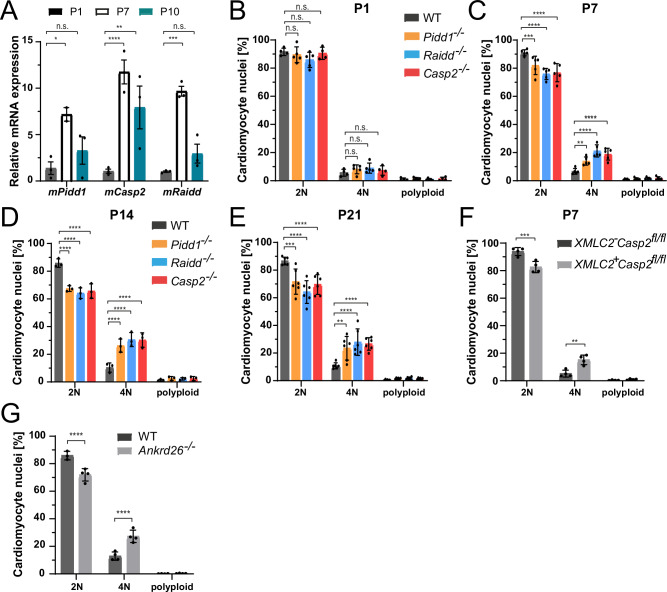
The PIDDosome controls CM ploidy levels starting on day 7 after birth. **A** PIDDosome mRNA levels were determined in isolated P1, P7 and P10 CMs from WT mice by qRT-PCR. n: 3 individual isolations per postnatal timepoint. Quantification of the CM nuclear ploidy measured by flow cytometry of P1 (**B**), P7 (**C**), P14 (**D**), P21 (**E**) from the indicated genotypes. n: 3–5 for P1 (5 hearts of each genotype per n), n: 5 for P7 (3 hearts of each genotype per n), n: 3 for P14 (2 hearts of each genotype per n), n: 4–6 for P21 (2 hearts of each genotype per n). **F** Quantification of the CM nuclear ploidy in *XMLC2*^*-*^*Casp2*^*fl/f*l^ and *XMLC2*^*+*^*Casp2*^*fl/fl*^ Mice at P7. n: 4 (*XMLC2*^*-*^*Casp2*^*fl/fl*^ and *XMLC2*^*+*^*Casp2*^*fl/fl*^) (n: 3 hearts of each genotype). **G** Quantification of CM nuclear ploidy of 3-month-old *Ankrd26*^*−/−*^ and WT (C57BL/6 J) mice measured by flow cytometry. n: 4 per genotype. Data represent means ± SEM (A) or ± SD (**B**–**G**) analyzed by Two-way ANOVA (**A**–**G**). *p < 0.05, **p < 0.01, ***p < 0.001, ****p < 0.0001, n.s. not significant.

To define the time window of PIDDosome action during postnatal CM development, we first evaluated postnatal mRNA expression of the individual PIDDosome components by qRT-PCR from isolated CMs. All PIDDosome components showed low expression levels at P1, while at P7 mRNA levels of all components were found to be strongly increased. Interestingly, decreasing expression of these genes at P10 suggests that the PIDDosome components are under tight transcriptional control during postnatal heart development (Fig. 3A). Next, in order to unveil when the PIDDosome exerts CM-specific ploidy control, hearts were isolated from WT as well as *Casp2*^*−/−*^, *Raidd*^*−/−*^ or *Pidd1*^*−/−*^ mice on P1, P7, P14 and P21 and CM nuclear ploidy was assessed by flow cytometry. As expected, on P1 no significant difference in the fraction of tetraploid CM nuclei was observed across genotypes (Fig. 3B), consistent with diploid mononucleated CMs dominating at this developmental stage [3]. However, at P7 the population of tetraploid CM nuclei in PIDDosome mutant mice increased significantly, compared to the same population in WT mice (Fig. 3C). This phenomenon coincides with the formation of binucleated CM at this time point during heart development [3]. Interestingly, the percentage of tetraploid CM nuclei reached a plateau at P14, as no further increases were noted on P21 (Fig. 3D, E), matching numbers were seen in adult mice (Fig. 1C). Flow cytometric ploidy analysis of *XMLC2*^*+*^*Casp2*^*fl/fl*^ mice confirmed that this is a cell-autonomous effect (Fig. 3F).

PIDDosome activation depends on PIDD1 binding to extra mother centrioles via the centriolar distal appendage protein ANKRD26 in cancer cells [22], as well as in primary hepatocytes [32]. Prior work has shown that rat P3 binucleated CMs contain 3 or 4 centrioles including clustered extra mother centrioles [16], suggesting these may be involved in pathway activation. Hence, we quantified the CM nuclear ploidy in hearts from 3-month-old mice lacking the molecule that links PIDD1 to mother centrioles, ANKRD26. Indeed, the percentage of tetraploid nuclei in *Ankrd26*^−/−^ mice was increased by two-fold, similar to what was observed in the PIDDosome knockout mice (Fig. 3G).

Our findings indicate that supernumerary centrosomes and specifically increased mother centriole numbers in CMs trigger PIDDosome activation in an ANKRD26-dependent manner as early as P7.

### Caspase-2-loss enhances gene expression signatures related to an immature CM state

In order to elucidate the biological processes through which the PIDDosome executes its ploidy-regulating function, P1, P7 and P14 CM nuclei were isolated from *XMLC2*^-^*Casp2*^*fl/fl*^ and *XMLC2*^+^*Casp2*^*fl/fl*^ mice and enriched by cell sorting based on PCM1 staining to perform bulk RNAseq (Supplementary Fig. 2B). Analyzing the transcriptomes of wild type CMs in a time-resolved manner by Gene set enrichment analyses (GSEA) supported data quality by confirming previously described developmental mRNA expression changes in CMs between P1, P7 and P14 [59, 60]. These included the previously reported  upregulation of different ion channels at P14, compared to P7 specimens, a known feature of CM maturation [61, 62]. In addition, gene sets linked to  cell adhesion were upregulated in P1 *vs.*  P7 CMs, consistent with previous publications  [60]. Gene sets of RNA splicing, aerobic respiration and oxidative phosphorylation were also abundant in P7 CMs in both comparisons, P14 *vs*. P7 and P7 *vs*. P1 samples, as clear signs of CM maturation [63]. Interestingly, when comparing the nuclear transcriptomes of CMs isolated from P7 *vs*. P1, gene sets defining “mitochondrial protein complexes” and “mitochondrial organization” were also enriched, indicating that P7 CMs have a more mature mitochondria structure or organization compared to P1 CMs, confirming published results [62, 64] (Supplementary Fig. 2C, D). In addition, a trend towards the switching of genes, which play a role in sarcomere structure and regulation, involved in the “Cardiac Fetal-to-Adult Contractile Isoform Transition” [60, 62], was also confirmed between P1 and P7 CMs (Supplementary Fig. 2E).

**Fig. 4:**
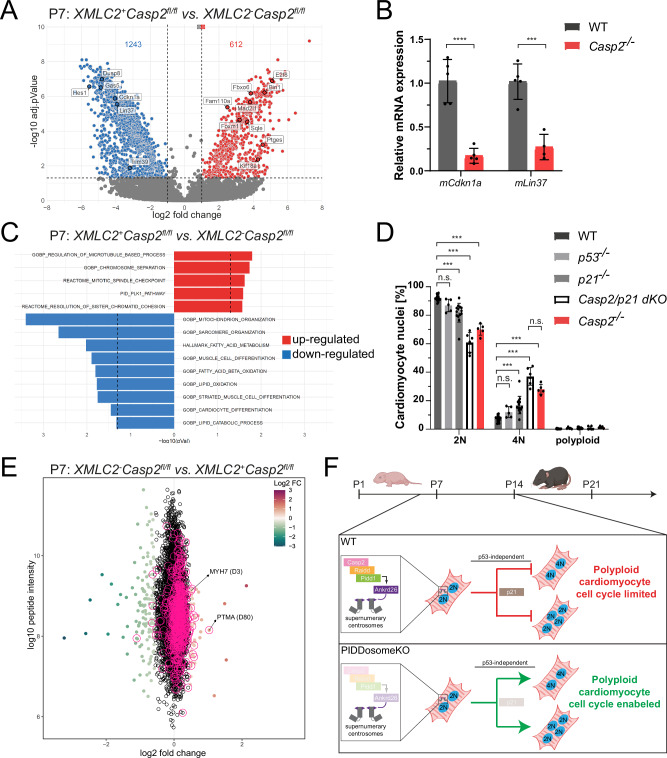
PIDDosome-mediated CM ploidy control does not depend on p53. **A** Volcano plot of the differential gene expression analysis comparing *XMLC2*^*+*^*Casp2*^*fl/fl*^ to *XMLC2*^*-*^*Casp2*^*fl/fl*^ samples at P7. Dashed lines indicate an adj. p-value of 0.05 and a log2FC of -1 or 1. Numbers of up- or down-regulated differentially expressed genes are indicated in the graph. **B** *p21 (Cdkn1a) and Lin37* mRNA levels in P7 CMs from WT and *Casp2*^*−/−*^ mice by qRT-PCR. n: 5-4 CM isolations per each genotype. **C** Selected significantly deregulated gene sets of the gene set enrichment analysis results comparing *XMLC*^*+*^ *Casp2*^*fl/fl*^ to*. XMLC2*^*-*^*Casp2*^*fl/fl*^ samples at P7. Dashed lines represent a plain *p*-value of 0.05. **D** Bar plots of CM nuclear ploidy of the indicated genotypes. n: 15 (WT), n:14 (*p21*^−/−^), n:5 (*p53*^−/−^), n: 6 (*Casp2*^*−/−*^*/p21*^*−/−*^), n:5 (*Casp2*^−/−^, as in Fig. 1C). **E** Plot showing the log2-fold change of N-termini measured by quantitative mass spectrometry in *XMLC2*^*-*^*Casp2*^*fl/fl*^ vs. *XMLC*^*+*^ *Casp2*^*fl/fl*^ heart tissues. Significantly up- or down-regulated N-termini (adjusted *p*-value  <  0.05) are color-coded based on the observed fold-change. N-termini generated by cleavage C-terminal to aspartic residues are highlighted with a purple outline. **F** Cartoon summary: The PIDDosome exerts its CM ploidy controlling function within the first two postnatal weeks in mice. Activation depends on extra centrosomes and ploidy is regulated independently of p53 stabilization, yet engaging p21 downstream of Caspase-2 in a yet to be defined manner. Data are mean ± SD (**B**–**D**) analyzed by Student’s *t* test (**B**) or Two-way ANOVA (**D**). ***p < 0.001, ****p < 0.0001, n.s. not significant.

Since the PIDDosome starts to affect the ploidy status of CMs during the first postnatal week (Fig. 3), we next investigated the differentially expressed genes in *Casp2*-depleted CMs (*XMLC2*^+^*Casp2*^*fl/fl*^ mice), compared to CMs from control animals (*XMLC2*^-^*Casp2*^*fl/fl*^ mice) at P7 (Fig. 4A and Supplementary Table S1). Interestingly, genes related to CM proliferation (*Foxm1* [65–67], *Gas5* [68, 69], *Sqle* [70], *Dusp8* [71]), cell cycle regulation (*Cdkn1a* [72], *Hes1* [73], *Trim39* [74], *Lin37* [75], *Kif18a* [76, 77]), ploidy control (*E2f8* [78], *Mad2l1* [79]) and T-Tubule maturation (*Bin1* [80]) were found differentially expressed between genotypes (Fig. 4A). qRT-PCR data of mRNA levels of *Cdkn1a and Lin37* confirmed the finding of the bulk RNAseq (Fig. 4B). Further, GSEA highlighted GO terms related to chromosome separation, mitotic spindle checkpoint, the PLK1 pathway (which also controls centrosome biogenesis), resolution of sister-chromatid cohesion and microtubule-based processes, all upregulated at P7 in *Casp2*-depleted CMs compared to controls. This suggests that Caspase-2-depleted CMs, which are already more polyploid compared to their control counterparts at P7, might experience mitotic errors and delays due to their increased DNA content. Importantly, downregulated GO terms in P7 *Casp2*-depleted CMs *vs*. P7 controls were associated with a reduction in fatty acid metabolism and muscle differentiation, as well as sarcomere organization, all signs of reduced CM maturation (Fig. 4C).

Together, this suggests that the absence of Caspase-2 in CMs causes delays in the naturally occurring terminal differentiation program, resulting in the presence of more immature and cell cycle active CMs, compared to control mice at P7.

### PIDDosome-driven CM ploidy control does not require *p53* but involves *p21*

Mechanistically, the PIDDosome regulates ploidy by the activation of the protease activity of Caspase-2 and subsequent MDM2 cleavage, leading to p53 activation [19, 23]. Thus, we analyzed CM ploidy in 3-month-old *p53*^−/−^ mice. Surprisingly, the percentage of tetraploid CM nuclei in mice lacking this key ploidy regulator was not significantly different compared to that found in WT animals (Fig. 4D). In liver development, p53 promotes a p21-induced cell cycle arrest in polyploid hepatocytes [23]. Intriguingly, *p21*^−/−^ mice showed a significant increase in ploidy, compared to WT animals but the percentage of 4 N nuclei in *p21*^−/−^ mice was still lower compared to that seen in the *PIDDosome* knockout mice (~16% vs. ~26%, respectively). This suggests that p21 and the PIDDosome may act in separate pathways (Fig. 4D). However, comparing CM nuclei from *Casp2*-deficient mice with those from *Casp2/p21* double-knockout (dKO) animals did not reveal an additional ploidy increase that was statistically significant, indicating that p21 acts downstream of the PIDDosome (Fig. 4D), as suggested also by our RNAseq data and qRT-PCR results (Fig. 4A, B). Since p73 has been implicated in regulating CM proliferation and can also be regulated by MDM2 in absence of p53 [81, 82], we reasoned that it may replace p53 during postnatal heart development. Thus, we analyzed the CM nuclear ploidy of *p73* knockout mice at P7, a time when the PIDDosome is already active. However, no difference in tetraploid CM population was evident between *p73*^*−/−*^ and WT mice (Supplementary Fig. 3C), indicating that p73 does not compensate for p53 during heart development, as seen in other developmental settings [83].

In an attempt to identify alternative substrates processed by Caspase-2 in response to PIDDosome activation in CMs, we performed N-terminomics with hearts isolated from *XMLC2*^*-*^*Casp2*^*fl/fl*^ and *XMLC2*^*+*^*Casp2*^*fl/fl*^ mice at P7 (Fig. 4E and Supplementary Fig. 3D). This analysis identified a total of 7503 unique neo-N-termini, of which 547 peptides resulted from a cleavage after aspartate residues, a hallmark of caspase-mediated proteolytic activity (Supplementary Table S2). Importantly, most of the cleavage events after aspartic residues were enriched in the control samples that express Caspase-2 (*XMLC2*^*-*^*Casp2*^*fl/fl*^) proving the validity of the approach. Among the putative Caspase-2 targets, two proteins were of interest: myosin heavy chain (MYH7), and Prothymosin alpha (PTMA). MHY7 is more abundant in fetal, compared to adult hearts [60, 62]. In the absence of Caspase-2, processed MHY7 is less abundant, suggesting that, in line with the observed transcriptomic changes (Fig. 4C), Caspase-2 loss delays CM maturation. Intriguingly, PTMA, the second substrate of interest, reportedly promotes CM proliferation [84] and Caspase-3-mediated proteolysis of PTMA has been noted in apoptotic cells [85].

Taken together, our findings support a role for p21 downstream of the PIDDosome that regulates CM ploidy independent of p53. If Caspase-2-mediated proteolysis of MHY7 and/or PTMA are indeed critical for ploidy control downstream of the PIDDosome needs to be addressed in future studies.

## Discussion

During early postnatal development, CMs lose their proliferative capability and become polyploid, which is an established barrier to heart regeneration [11, 12]. Here, we show that the PIDDosome multiprotein complex is critical for regulating CM polyploidization within the first two postnatal weeks. Mice lacking individual PIDDosome components exhibit an increased nuclear and cellular ploidy status in CMs, which does not alter cardiac structure nor function in early adulthood, but appears to gradually compromise cardiac function with age (Fig. 4F). A recent study suggests that CM ploidy is necessary for proper organ function, as an increase in mononucleated diploid (and smaller) CM cause a decrease in the EF in mice [86]. Conversely, increased ploidy should increase the EF. The lack of a clear correlation between increased ploidy and CM size observed in young PIDDosome-deficient mice might explain why cardiac function is not altered at a young age. Of note, the observed reduced cardiac output in aged animals requires a more detailed follow-up analysis. If the loss of PIDDosome function may affect tissue performance after myocardial infarction (MI) still needs to be tested. However, it was shown that transient overexpression of Plk1 and Ect2 in polyploid CMs promotes CM proliferation and cytokinesis, leading to increased CM numbers. Importantly, this strategy improved heart function post-MI [86]. Hence, cell cycle re-entry of polyploid CMs may facilitate regeneration. Thus, we propose that suppression of PIDDosome function, e.g. by Caspase-2 inhibition [87–89], might be beneficial for cardiac regeneration after heart injury.

In contrast to the liver [23], in CMs the PIDDosome limits polyploidy by modulating different cell cycle genes, including p21, independently of p53 activity. Remarkably, CyclinG1, a p53 target gene, was shown to affect ploidy levels in the heart when overexpressed in neonatal rat CM, while its loss in mice prevented ploidy increases in response to pressure overload [90]. However, while p53 has been repeatedly implicated in CM death in response to DNA damage [91, 92], a direct impact on CM development has never been reported. How Caspase-2 can engage p21 in the absence of p53 is unclear, but stabilizing effects of Caspase-2 on p21 mRNA translation have been reported [93]. Regardless of mechanism, these data together with the increased percentage of 4N CM nuclei in p21 knockout animals suggest that the PIDDosome might exert its function not exclusively via p21, as the increase in 4N CM nuclei in p21 knockout mice was significantly lower than that seen in PIDDosome-deficient CMs. These findings are in line with previous studies which have shown that postnatal CM proliferation depends on p21 [94] but not on p53, as a *Trp53* knockout is insufficient to induce CM proliferation [95].

E2F8 is known to be a fundamental and positive regulator of hepatocyte ploidy during liver development [78] where it transcriptionally targets *Casp2* and *Pidd1* [23]. Recently, it was reported that CM-specific double knockout of *E2f8*  and in mice increases the  percentage of mononucleated diploid CMs, which had no effect on cardiac function in steady state and also failed to improve heart regeneration after infarction [96]. Our bulk nuclear RNAseq data has shown that cardiac-specific deletion of Caspase-2 correlates with an upregulation of *E2f8* expression, which could explain the increased level of polyploid CMs in these mice [96]. *E2f7/8* double knockout livers are characterized by almost exclusively mononucleated diploid hepatocytes, while in the heart the inactivation of *E2f7/8* causes a modest increase of mononucleated diploid CMs from ~1% to 5% [96]. Thus, comparing liver and heart development, it seems that there are additional mechanisms or Caspase-2 substrates, which restrict CM polyploidization during heart development aiming to secure steady state heart function. Intriguingly, PTMA, identified in our N-terminomics analysis has been reported to enforce STAT3 signaling during CM proliferation [84]. Moreover, Caspase-3-mediated proteolysis of PTMA has been noted in apoptotic cells [85], but if Caspase-2 indeed processed PTMA to alter its function remains to be established. Of note, we identified D80 as a possible caspase-2 cleavage site. Caspase-2, −3 and −7 can cleave PTMA at D7 when spiked into cell lysates, while Caspase-3 reportedly targets D99 during apoptosis [85, 97]. Hence, we need to interpret our results with caution, in particular since  other proteases can also cleave proteins after aspartate residues.

Finally, heart regeneration therapies based on CM proliferation face many obstacles in clinical studies.  Hence, dissecting the events that regulate polyploid CM proliferation during postnatal heart development will be essential for translational medicine. As such, the PIDDosome represents an attractive target to modulate cell cycle arrest in human polyploid CMs, considering recent finding documenting improved post-MI function of hearts overexpressing PLK1 and ECT2 [86]. However, it is important to acknowledge that human adult hearts consist of different polyploid CM populations, while in mice the majority is binucleated with two diploid nuclei [3]. This hints towards possible species-specific differences in regulating CM polyploidization that deserve further interrogation. Thus, it remains possible that the role of the PIDDosome in CMs is not as prominent in humans.

To conclude, our findings not only describe a critical regulatory step in the terminal differentiation program activated postnatally in CMs, but also open a new perspective for regenerative therapies based on polyploid CMs, instead of focusing on the limited regenerative potential of the few mononucleated CM present in the mammalian heart.

## Supplementary information

Supplemental Figure and Table legends

Suppl. Figs. 1-4

qPCR raw data

qPCR raw data

qPCR raw data

DEG p7 wt vs. C2 KO

N-tails Substrate Analysis

## Data Availability

Data supporting the findings of this study are available within the paper and its Supplementary Information. Additional data that support the findings of this study are available from the authors upon reasonable request. Information on data base repositories for RNAseq and N-tails proteomics are found in the materials section.
