# Supplementary material for: The PIDDosome controls cardiomyocyte polyploidization during postnatal heart development
Source: Cell Death Differ. 2026 Jan 12;33(6):1292–304. doi: 10.1038/s41418-025-01645-x (PMC13246752; doi:10.1038/s41418-025-01645-x)
Supplement: Supplementary file 1 — Supplemental Figure and Table legends [file 41418_2025_1645_MOESM1_ESM.docx]

**Supplemental Legends**

**Supplemental Figure 1. CRE expression under control of the *XMLC2* promoter does not affect CM nuclear ploidy.**

(**A**) Schematic representation of the flow cytometry gating strategy used to assess CM nuclear ploidy. Nuclei were isolated from frozen hearts and CM nuclei were identified as PCM1^+^. The DNA content was measured by PI staining (**B-C**) Schematic illustrations of the mouse gene loci modified to induce CM-specific deletion of *Casp2* (B) and CM-specific membrane labelling (mT/mG) driven by *XMLC2*-dependent Cre activation (C). (**D**) Quantification of CM nuclear ploidy of the indicated genotypes measured by flow cytometry. *XMLC2^-^* (n=3), *XMLC2^+^*(n=4). (**E**) Representative H&E stained FFPE heart sections of 3 months-old mice of the indicated genotypes. Scale bars: 1mm. (**F**) Quantification of cardiac functions, left ventricle volume diastole and systole (LV Vol;d and LV Vol;s), left ventricular internal diameter end diastole and end systole (LVID;d and LVID;s), left ventricle posterior wall diastole and systole (LVPW;d and LVPW;s), interventricular septum diastole and systole (IVS;d and IVS;s)) measured by echocardiography in 7-10-weeks-old mice. n: 21 (WT), n: 18 (*Pidd1*^-/-^). (**G**) Quantification of cardiac functions, left ventricle volume diastole and systole (LV Vol;d and LV Vol;s), left ventricular internal diameter end diastole and end systole (LVID;d and LVID;s), left ventricle posterior wall diastole and systole (LVPW;d and LVPW;s), interventricular septum diastole and systole (IVS;d and IVS;s) measured by echocardiography in 15-19-months-old (aged) mice. n: 11 (WT), n: 13 (*Pidd1*^-/-^). (**H**) Representative FFPE heart sections of 15-19-months-old (aged) mice stained for collagen. Scale bars: 100µm. Data represent means ± SD (D, F-G) analyzed by Student’s t test (F-G) or Two-way ANOVA (D). * p < 0.05, **p < 0.01, ***p < 0.001 n.s.: not significant.

**Supplemental Figure 2. Gene set enrichment analysis results of control CMs supports the previously described developmental changes between P1, P7 and P14 CMs.**

(**A**) Representative images of immunohistochemical analysis of cell death by TUNEL staining of heart sections of indicated genotype. Irradiated small intestine sections were used as positive controls. Scale bars: 20 µm. (**B**) RLDF (regularized linear discriminant functions) representation of the CM RNAseq samples, based on the centered, log2-transformed counts-per-million (log2CPM) (note: before count normalization with limma). The sample groups as indicated in the legend were defined as RLDF training groups. RDLF was performed with the function ‘plotRLDF’ of the R package limma (v. 3.52.4). (**C**-**D**) Selected significantly deregulated gene sets of the gene set enrichment analysis results comparing developmental days in control mice (*XMLC2^-^Casp2^fl/fl^*). Dashed lines represent a plain p-value of 0.05. (**E**) Heatmap of selected genes involved in sarcomere structure and regulation. The Z-scores of normalized gene expression calculated across P1 and P7 control samples are shown.

**Supplemental Figure 3. Bulk RNA sequencing sample exclusion**

(**A**) PCA of the CM RNAseq samples, based on the centered, log2-transformed counts-per-million (log2CPM) (note: before count normalization with limma). The samples are labelled with their respective group and replicate number; for improved readability of the labels, ‘*XMLC2^-^ Casp2^fl/fl^*’ was replaced with ‘minus’ and ‘*XMLC^+^ Casp2^fl/fl^*’ was replaced with ‘plus’. (**B**) Top 20 enriched gene sets (by adjusted p-value) of fuzzy clusters 6 and 7 genes determined by functional enrichment analysis. (**C**) Quantification of the CM nuclear ploidy of *p73^-/-^* mice measured by flow cytometry at P7. n:4 (WT and *p73^-/-^*). Data are mean ± SD analyzed by Two-way ANOVA; n.s.: not significant. (**D**) Principal component analysis based on log2 transformed N-terminal peptide intensities.

**Supplemental Figure 4. Fuzzy clustering of all samples**

Fuzzy clustering of all CM RNAseq samples. Clustering was performed on the gene log2 fold changes with respect to the average gene counts of the four P1 *XMLC2^-^ Casp2^fl/fl^* replicates, after each sample’s raw counts were normalized with the average count of its interquartile range count values.

**Table S1:** Differentially expressed genes in *Casp2*-depleted CMs (*XMLC2^+^Casp2^fl/fl^* mice), *vs.* CMs from control animals (*XMLC2^-^Casp2^fl/fl^* mice) at P7.

**Table S2:** Neo-N-termini identified in hearts isolated from *XMLC2^-^Casp2^fl/fl^* and *XMLC2^+^Casp2^fl/fl^* mice on P7.
