# Supplementary figures and images for: The PIDDosome controls cardiomyocyte polyploidization during postnatal heart development
Source: Cell Death Differ. 2026 Jan 12;33(6):1292–304. doi: 10.1038/s41418-025-01645-x (PMC13246752; doi:10.1038/s41418-025-01645-x)

# Leone M *et al.* Suppl Figure 1

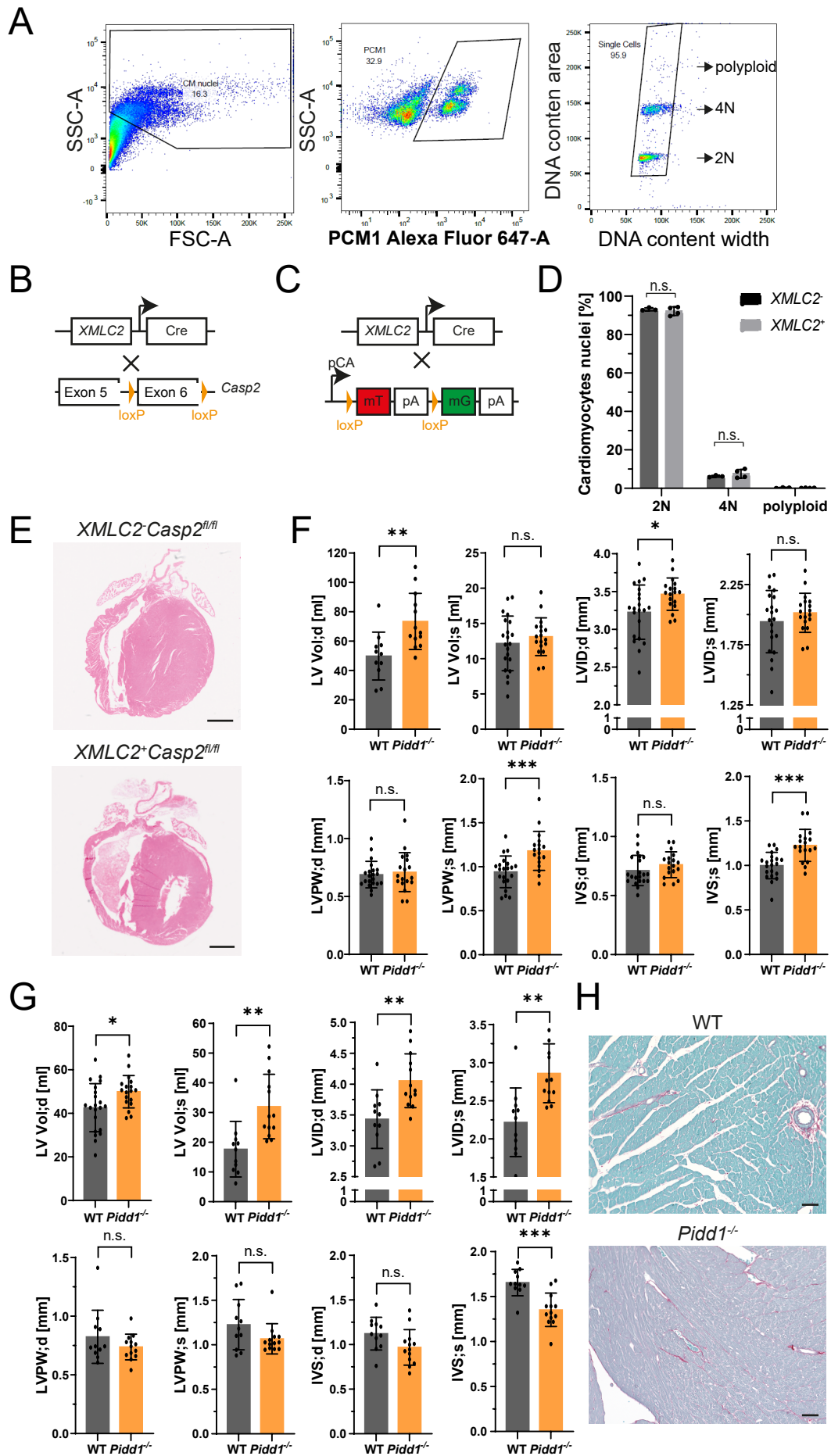

Leone M *et al.* Suppl Figure 2

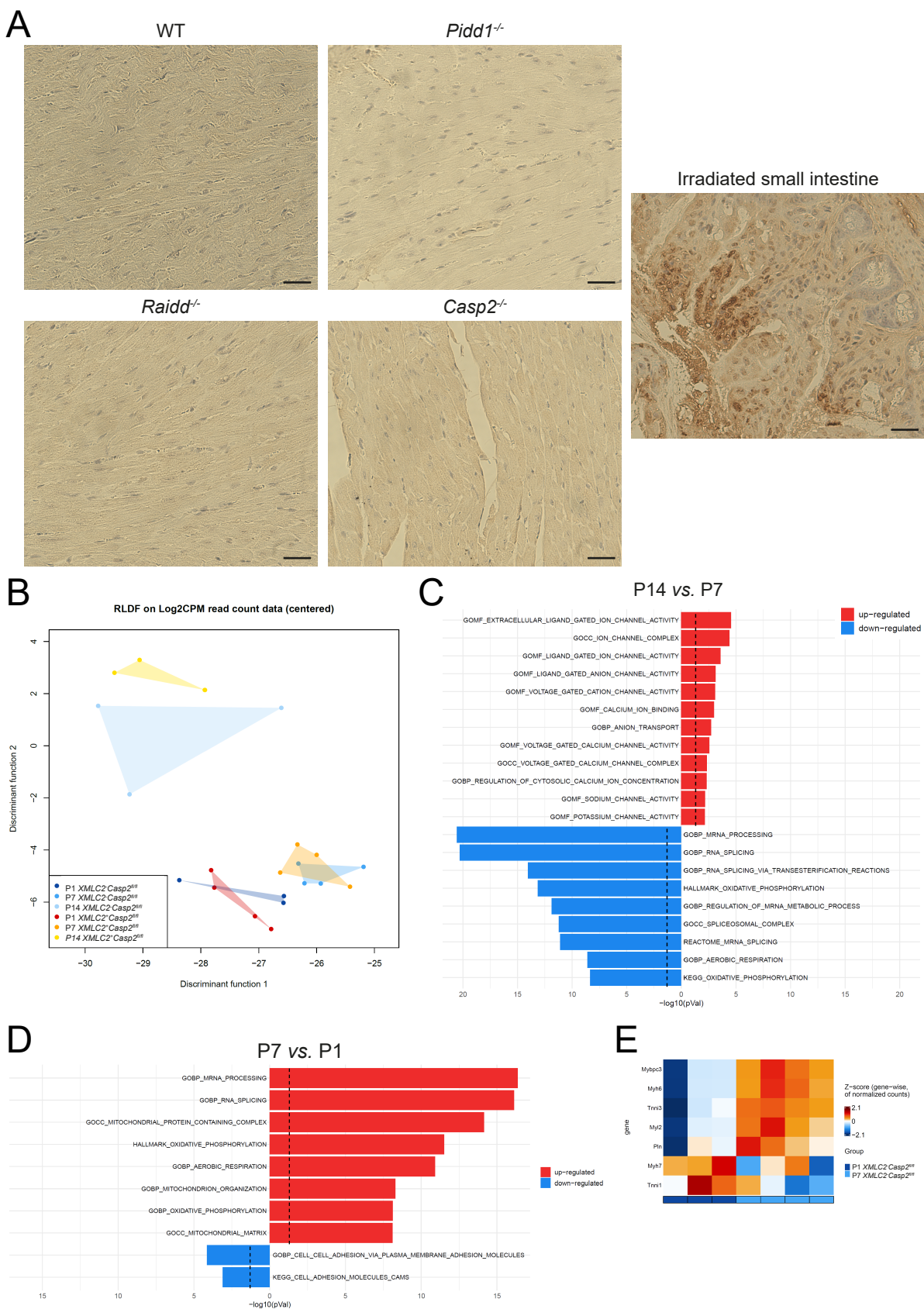

Leone M *et al.* Suppl Figure 3

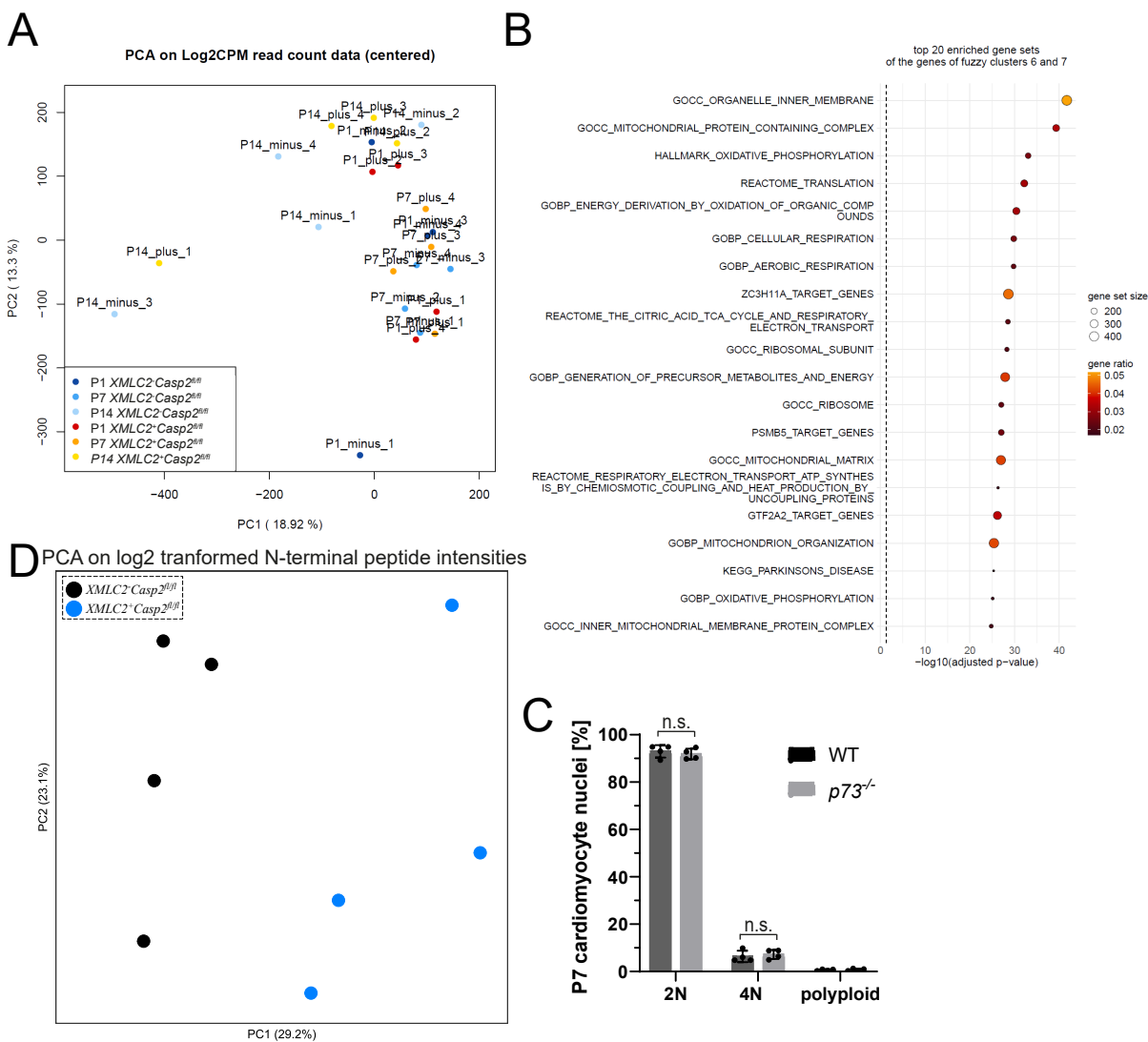

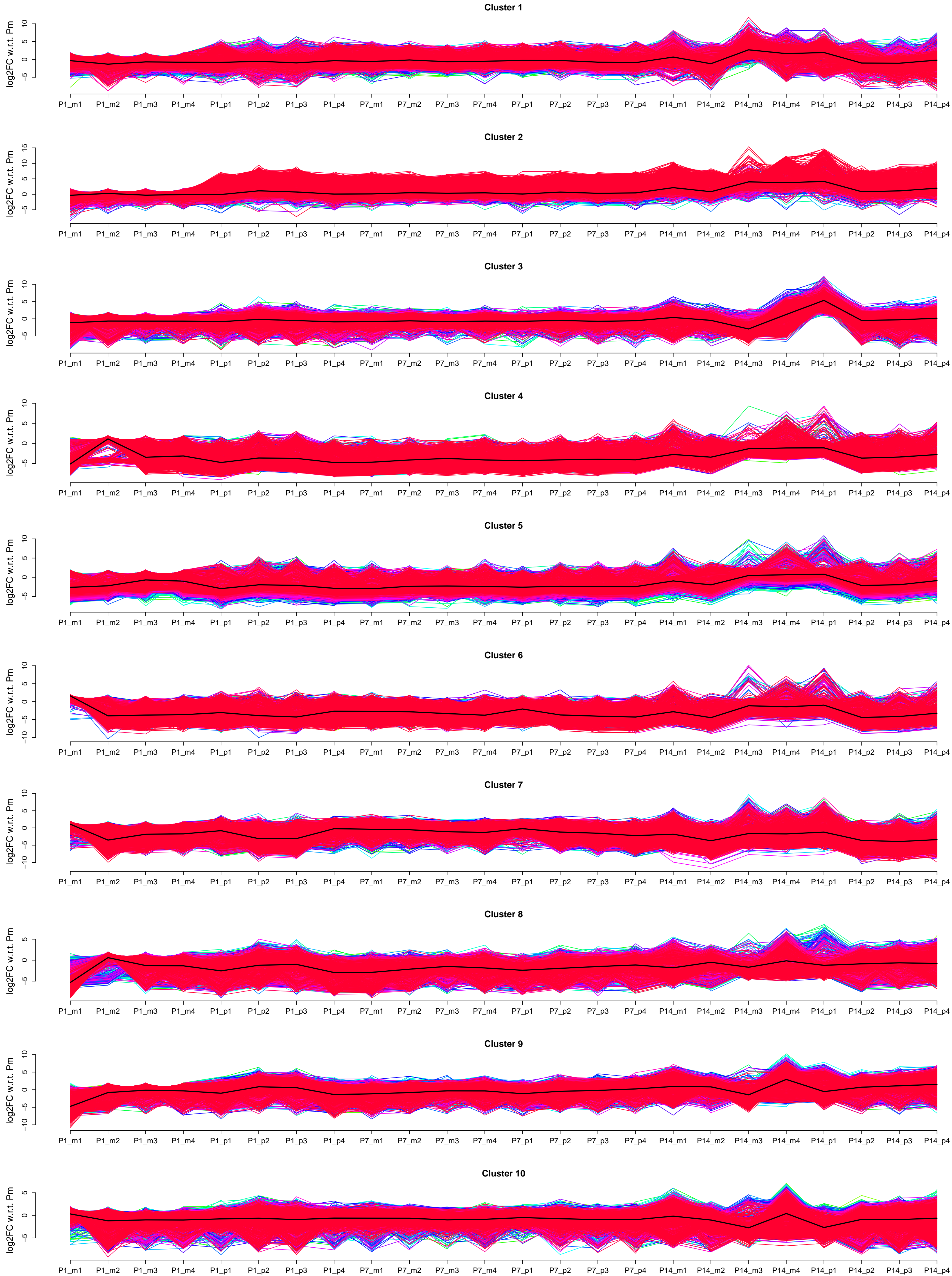

Supplement: Supplementary file 2 — Suppl. Figs. 1-4 [file 41418_2025_1645_MOESM2_ESM.pdf]
